# Photoacoustic and modulated reflectance studies of indirect and direct band gap in van der Waals crystals

**DOI:** 10.1038/s41598-017-15763-1

**Published:** 2017-11-13

**Authors:** Szymon J. Zelewski, Robert Kudrawiec

**Affiliations:** Department of Experimental Physics, Faculty of Fundamental Problems of Technology, Wroclaw University of Science and Technology, Wybrzeże Wyspiańskiego 27, 50-370 Wrocław, Poland

## Abstract

Photoacoustic (PA) and modulated reflectance (MR) spectroscopy have been applied to study the indirect and direct band gap for van der Waals (vdW) crystals: dichalcogenides (MoS_2_, MoSe_2_, MoTe_2_, HfS_2_, HfSe_2_, WS_2_, WSe_2_, ReS_2_, ReSe_2_, SnS_2_ and SnSe_2_) and monochalcogenides (GaS, GaSe, InSe, GeS, and GeSe). It is shown that the indirect band gap can be determined by PA technique while the direct band gap can be probed by MR spectroscopy which is not sensitive to indirect optical transitions. By measuring PA and MR spectra for a given compound and comparing them with each other it is easy to conclude about the band gap character in the investigated compound and the energy difference between indirect and direct band gap. In this work such measurements, comparisons, and analyses have been performed and chemical trends in variation of indirect and direct band gap with the change in atom sizes have been discussed for proper sets of vdW crystals. It is shown that both indirect and direct band gap in vdW crystals follow the well-known chemical trends in semiconductor compounds.

## Introduction

Van der Waals (vdW) crystals are known for a long time^1–14^ but in recent years some of them have gained significant interest because of unique mechanical and optical properties of samples obtained by exfoliation of bulk material to atomically thin layers^15–25^. Since pioneering reports on optical properties of MoS_2_ layers^15,16^, it is well established that the electronic band structure of MoS_2_ strongly varies with the number of layers and exhibits indirect-to-direct band gap transition with the size reduction to a single layer. Similar studies of optical properties and the electronic band structure have been performed on other van der Waals crystals such as MoSe_2_, MoTe_2_, WS_2_, and WSe_2_^22–25^. Some of them were investigated quite recently as bulk materials^13,14^ but interest in these materials was low due to no potential applications in semiconductor devices. At this moment the situation is different. VdW crystals are exciting because of interesting physics of two-dimensional (2D) layers and their potential applications in novel semiconductor devices^26–28^. Because of this even bulk van der Waals crystals are interesting to study since many of them are not explored experimentally. One method which has never been applied to study most of van der Waals crystals is photoacoustic (PA) spectroscopy. We show that this technique is an excellent tool to study the band gap in van der Waals crystals since it is sensitive to indirect gap transitions which cannot be observed in photoluminescence. In combination with modulated reflectance (MR), which is known as a state of the art absorption-like technique to study direct optical transitions^29^, it is possible to conclude about the band gap character in van der Waals crystals and the spectral separation between the indirect and direct gap. In this work we applied PA and MR to study indirect and direct gap in many van der Waals crystals including dichalcogenides (MoS_2_, MoSe_2_, MoTe_2_, HfS_2_, HfSe_2_, WS_2_, WSe_2_, ReS_2_, ReSe_2_, SnS_2_, and SnSe_2_) and monochalcogenides (GaS, GaSe, InSe, GeS, and GeSe).

**Figure 1:**
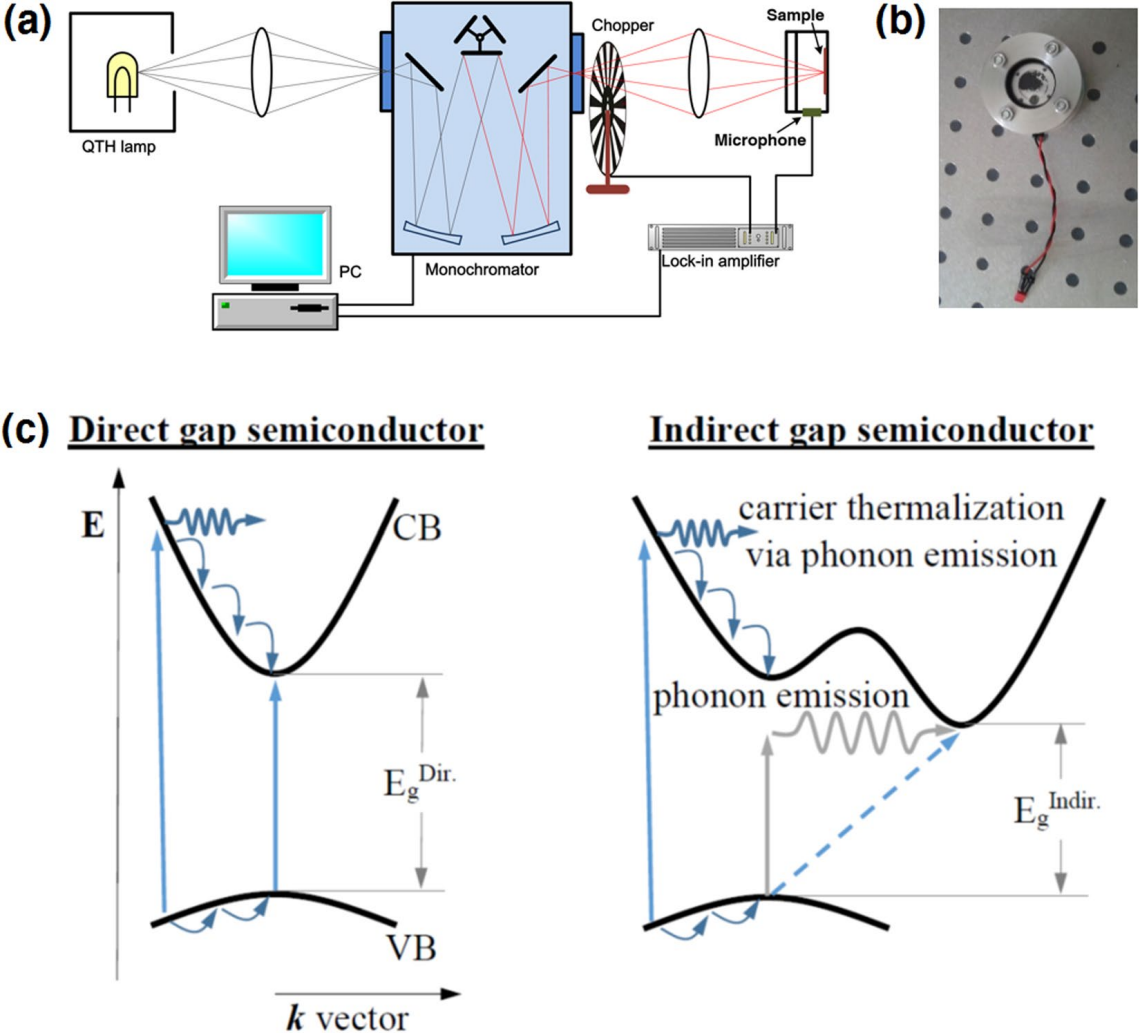
A sketch of experimental setup for photoacoustic spectroscopy (**a**), a photo of the photoacoustic cell (**b**), and a sketch of light absorption and phonon generation in an indirect gap semiconductor (**c**).

Figure 1 shows the concept of PA measurements with significant details in measuring apparatus and principles of PA signal generation. A monochromatic light (in this case the light from a halogen lamp dispersed by a monochromator) is modulated at a given frequency and focused onto the sample which is enclosed in an air-tight cell, see the photoacoustic cell in Fig. 1b. It is worth noting that no special sample preparation for PA measurements is required in this case. Due to absorption of light inside the sample non-radiative processes occur and cause heat generation, i.e., a temperature variation. The periodically generated heat is transferred to the gas of the cell due to thermal conduction and causes pressure oscillations at the modulation frequency. This oscillation is detected by an acoustic transducer with a lock-in amplifier. In general, the amplitude of PA signal depends on the amount of generated heat which is strongly correlated with the optical absorption coefficient. The intensity of PA signal, which is related to heat generation inside the sample, is unmeasurable when a very weak light absorption is present inside the sample, and the detection limit is determined by the parasitic signal produced from PA cell walls, transmission window or various contaminants. However, for indirect gap semiconductors the heat generation is strong even if the absorption coefficient is weak. The reason for that is the phonon generation necessary to fulfil the momentum conservation during the light absorption, see the sketch in Fig. 1c. This is an important advantage of PA spectroscopy over regular absorption measurements (combined transmission and reflectance measurements) where the determination of the absorption edge for indirect semiconductors becomes difficult if the sample thickness is small and/or irregular, i.e., the case of exfoliated van der Waals crystals. The PA signal is proportional to the optical absorption coefficient until it saturates in the high absorption region, in case of semiconductor materials above the fundamental band gap. Therefore, measurements of PA signal intensity as a function of incident photon energy allow to determine the absorption edge in semiconductor samples of either direct or indirect band gap.

So far, PA spectroscopy was widely applied to study various semiconductor materials^30–37^ but most of van der Waals crystals remain unexplored by this method. PA spectroscopy can be used to determine the band gap as shown in refs^31,34,37^ as well as the impurity and defect-related absorption^33,35^. Moreover, this technique can be used to study the surface and bulk nonradiative recombination^32^ and the thermal diffusivity^36^. Very important advantage of this method is that it can be applied even if the investigated samples are very small or the incident light is scattered due to sample surface irregularities, since only the absorbed light fraction causes the signal generation. We recently utilized this advantage to study GaAsBi nanowires^38^ but it is also worth highlighting in the case of van der Waals crystals.

In contrast to PA spectroscopy, the MR technique (photoreflectance (PR), contactless electroreflectance (CER), and piezoreflectance (PzR)) probes exclusively direct optical transitions which are present at singularities of joint optical density of states^39^. For direct gap semiconductors the optical transition with the lowest energy is the band gap transition, but for indirect gap semiconductors the direct optical transitions are observed in MR spectra at energies larger than the fundamental band gap. So far MR spectroscopy was applied few times to study van der Waals crystals^12,39–46^ but many of them were not studied yet by this technique. In this work we use MR spectroscopy as a complementary method to study the band gap in van der Waals crystals, which allows to determine the character of band gap observed in PA spectra. We show that by comparing PA and MR spectra it is possible to conclude about the band gap character in van der Waals crystals.

## Results and Discussion

**Figure 2:**
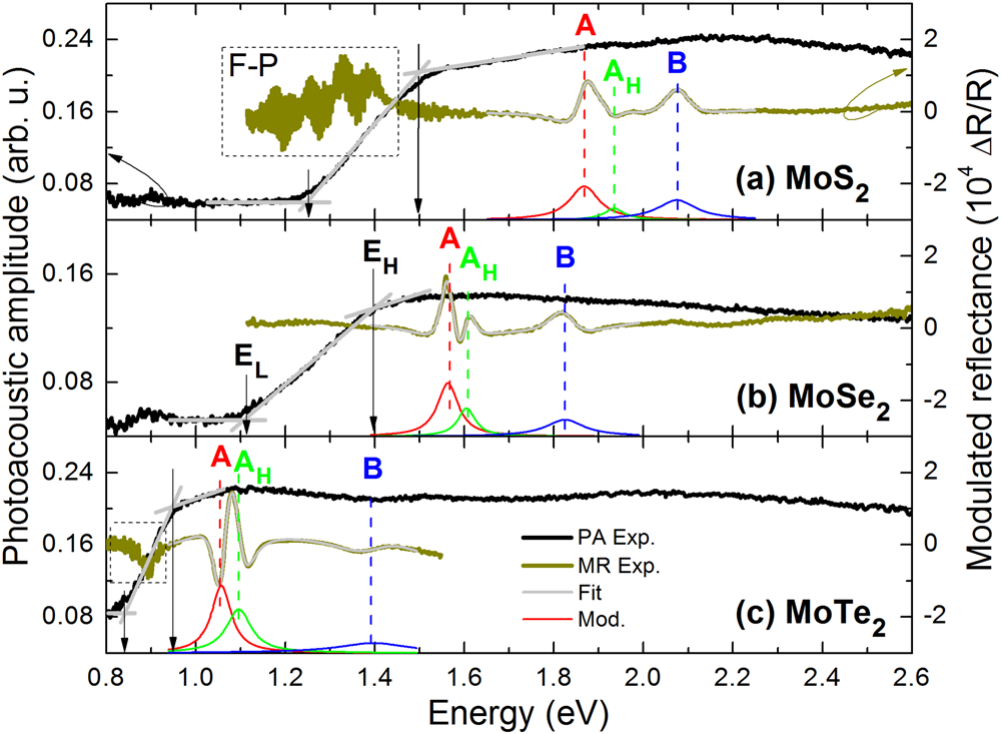
Room temperature photoacoustic spectra (black line) and modulated reflectance spectra (dark green line) of (**a**) MoS_2_, (**b**) MoSe_2_, and (**c**) MoTe_2_ crystals. The PA signal at ~0.9 eV is associated with light absorption by H_2_O molecules in the air-tight PA cell.

**Figure 3:**
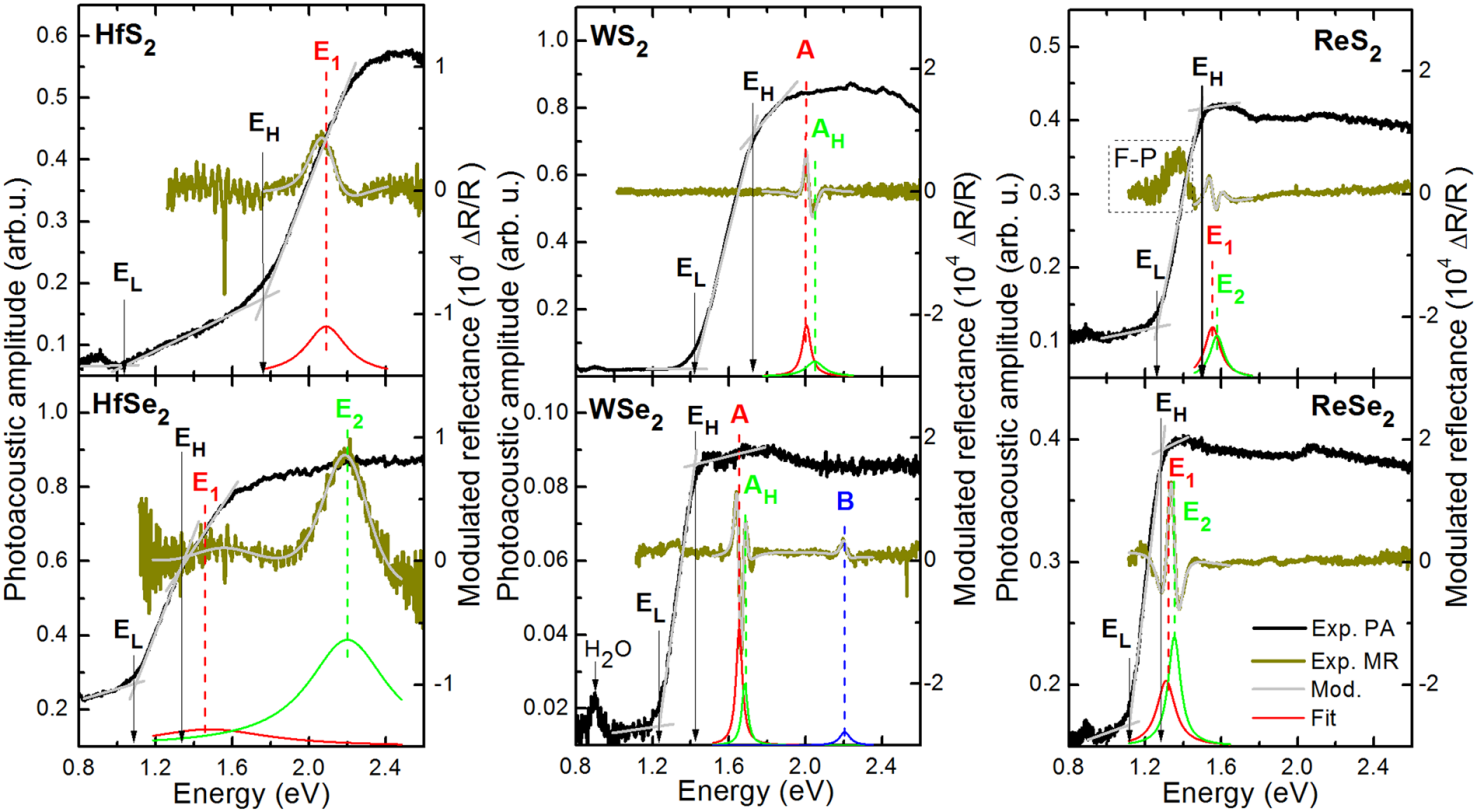
Room temperature photoacoustic spectra (black line) and modulated reflectance spectra (dark green line) of (**a**) HfS_2_, (**b**) HfSe_2_, (**c**) WS_2_, (**d**) WSe_2_, (**e**) ReS_2_, and (**f**) ReSe_2_ crystals.

Figures 2 and 3 show PA and MR spectra measured at room temperature for transition metal dichalcogenides (MoS_2_, MoSe_2_, MoTe_2_, HfS_2_, HfSe_2_, WS_2_, WSe_2_, ReS_2_, and ReSe_2_). So far most of them (MoS_2_, MoSe_2_, WS_2_, WSe_2_, ReS_2_, and ReSe_2_) were studied by MR (photoreflectance, electroreflectance or piezoreflectance)^12,39–41,43–46^, but none of them was studied by PA spectroscopy. In order to discuss chemical trends for the indirect and direct gap in these materials, they are grouped in a few sets of samples going from smaller to larger atoms. In this case MoX_2_ with X = (S, Se, Te) represents transition metal dichalcogenides (TMDs) with the smallest atom from the group of transition metals. Next, couples of YS_2_ and YSe_2_ samples with Y = (Hf, W, Re) are shown and discussed.

For samples shown in Figs 2 and 3 no photoluminescence is observed even at low temperatures (15 K) because of the indirect fundamental gap character in these compounds, as confirmed by comparing PA spectra with MR spectra. For each sample shown in Figs 2 and 3 the PA signal increases above the background with the increase in photon energy and saturates at energies much smaller than the energy of MR resonance which corresponds to the direct band gap.

To determine the absorption edge from PA measurements, the so-called knee method can be applied^37^. This method takes into account the intersection between two tangent lines in the PA saturation region (see proper lines in Figs 2 and 3), and thereby neglects a weak absorption which is related to the indirect gap as well the Urbach tail in the total optical density of states. This way, the band gap estimation is based on the spectrum shape analysis from the high energy side. On the other hand, we see that the PA signal also appears at significantly lower energies due to the weak indirect gap absorption and the Urbach tail. Therefore, we determine two characteristic edges present in PA spectra, first one by applying the knee method (labeled E_H_), and second one in a similar manner from the low energy side (labeled E_L_). The energy separation between E_H_ and E_L_ can be treated as an indicator of the sample quality within a given compound. For samples with large tails in the density of states this separation can be significant while for high quality samples it should reflect the band gap thermal broadening. It means that the middle between E_L_ and E_H_ corresponds to the band gap for samples with negligible Urbach tails. For samples studied in this paper both E_L_ and E_H_ are extracted from PA spectra. The band gap position is determined as ${E}_{g}^{PA}=({E}_{L}+{E}_{H})/2$ and its broadening as ${\Gamma }_{g}^{PA}=({E}_{H}-{E}_{L})/2$. Values of band gap and their broadening determined in this way for the studied samples are listed in Table 1. In addition, the direct gap energy and its broadening extracted from MR spectra together with the bandgap and its broadening determined from PA spectra for the studied samples are listed in Table 1. The accuracy of *E*_*L*_ and *E*_*H*_ determination is estimated graphically to be below 5 meV. It means that the ${E}_{g}^{PA}$ and ${\Gamma }_{g}^{PA}$ is determined from PA measurements with the accuracy better than 10 meV. The accuracy of the band gap determination from MR measurements $({E}_{g}^{MR})$ is taken from the fitting procedure to be below 5 meV while the accuracy of broadening parameter $({\Gamma }_{g}^{MR})$ is below 2 meV.

**Table 1:** Band gap transition values and their broadenings determined from PA (E_g_^PA^ and Γ_g_^PA^) and MR (E_g_^MR^ and Γ_g_^MR^) measurements at room temperature together with the literature data of indirect (E_g_^Indir.^) and direct (E_g_^Dir^) band gap.

Compound	E_g_^PA^ (eV)	Γ_g_^PA^ (eV)	E_g_^MR^ (eV)	Γ_g_^MR^ (eV)	ΔE (eV)	E_L_ (eV)	E_g_ Eq.(3) (eV)	E_g_^Indir^. (eV)	E_g_^Dir.^ (eV)	Cryst. Structure
MoS_2_	1.37	0.12	1.87	0.04	0.50	1.25	1.19	~1.3^*^	1.87^34^	2H polytype
MoSe_2_	1.25	0.14	1.56	0.0	0.31	1.11	1.01	~1.1^**^	1.56^34^	
MoTe_2_	0.89	0.06	1.06	0.027	0.17	0.83	0.80	~0.8^*^	1.15^25***^	
HfS_2_	1.39	0.26	2.09	0.131	0.70	1.03	1.00	1.96^44^	NA	1T polytype
HfSe_2_	1.21	0.13	1.49	0.380	0.28	1.08	0.96	1.19^44^	NA	
WS_2_	1.57	0.15	2.05	0.027	0.48	1.42	1.30	~1.3^**^	2.05^33^	2H polytype
WSe_2_	1.33	0.09	1.65	0.020	0.32	1.24	1.16	~1.3^**^	1.70^33^	
ReS_2_	1.37	0.11	1.55	0.049	0.18	1.26	1.20	1.52^36**^	1.554^36**^	3R polytype
ReSe_2_	1.18	0.06	1.31	0.058	0.03	1.12	1.06	1.36^36**^	1.387^36**^	
SnS_2_	2.36	0.23	NA	NA	NA	2.13	2.00	~2.2^**^	NA	2H polytype
SnSe_2_	1.24	0.10	NA	NA	NA	1.14	1.06	NA	NA	
GaS	2.87	0.37	2.92	0.390	0.05	2.50	2.26	2.53^40^	2.9^40^	2H polytype
GaSe	1.98	0.17	1.98	0.032	~0	1.81	1.93	NA	~2.0^*^	
InSe	1.23	0.07	1.22	0.022	−0.01	1.16	1.21	NA	1.25^53***^	
GeS	1.60	0.11	1.64	0.033	0.04	1.49	1.45	1.6^57^	NA	Orthorhombic
GeSe	1.22	0.11	NA	NA	NA	1.11	1.00	1.2^57^	NA	

ΔE is the energy difference between the band gap transition determined from MR an PA measurements. E_L_ is the low energy absorption edge determined from PA measurements. E_g_ is the absorption edge determined from PA measurements and the application of Eq. (3) to analyze these spectra, see Fig. 7.

^*^2D Semiconductor web page, ^**^HQ Graphene web page, ^***^low temperature.

The indirect gap for MoS_2_, MoSe_2_, and MoTe_2_ has been determined from PA spectra to be 1.37, 1.25, and 0.89 eV, respectively, while the direct gap determined from MR spectra is 1.87, 1.56, and 1.06 eV, respectively. It gives the energy separation of 0.50, 0.31, and 0.17 eV between the direct and indirect gap in MoS_2_, MoSe_2_, and MoTe_2_, respectively. In MR spectra besides the A transition, which corresponds to the direct gap at the K point of Brillouin zone, A_H_ and B transitions are observed^39^ and fitted by the Aspnes formula. The A_H_ transition is attributed to the direct optical transition at the H point of Brillouin zone, which is very symmetric and similar to the electronic band structure at the K point, while the B transition is attributed to the optical transition between the spin-orbit split-off band and the conduction band at the K point of Brillouin zone. An analogous B_H_ transition is expected near B transition but is not resolved in MR spectra due to small energy separation between these transitions^39^. Energies of direct optical transitions in MoS_2_ and MoSe_2_ are in a good agreement with the previous MR studies^39^. The bulk MoTe_2_ was not studied yet by MR spectroscopy but the direct optical transition observed for this crystal is very consistent with recent reflectance studies of layered MoTe_2_ in the bulk regime^25^. Since the Ubrach tail significantly influences the determination of absorption edge for indirect semiconductors and can vary from sample to sample, this energy is more difficult to compare with other studies and the literature. However, it has been found that the indirect band gap determined from PA measurements is consistent with other reports, see Table 1.

The studied MoS_2_, MoSe_2_, and MoTe_2_ samples belong to the hexagonal (2H) polytype layered crystals. In the plane strong covalent-ionic bonds dominate while the bonding between the dichalcogenide layers is van der Waals-like. For this reason, these crystals can be regarded as quasi two dimensional. The configuration of the transition metal Mo is 5s^1^4d^5^ and that of the chalcogenides S, Se, and Te is 3s^2^3p^4^, 4s^2^4p^4^, and 5s^2^5p^4^, respectively. Electrons from orbitals of the same symmetry (s and p) but different shells are responsible for the change is in the electronic band structure in this set of samples. With the increase in the shell number the lattice constant increases, leading to a reduction of band gap difference as well as absolute values of direct and indirect band gaps. The observed chemical trend for indirect and direct gap is similar to the one known in III-V and II-VI semiconductors. By replacing small atoms by larger atoms the lattice constant increases and the band gap narrows.

Very similar trends are observed for remaining TMD samples shown in Fig. 3. In this case we can investigate the influence of chalcogenide atoms (compare rows in Fig. 3) as well as transition metal atoms (compare columns in Fig. 3) on the optical properties of these materials.

We start the analysis from the influence of chalcogenide atoms on the indirect and direct band gap. The indirect gap for HfS_2_ and HfSe_2_ has been determined from PA spectra to be 1.39 and 1.21 eV, respectively, while the direct gap determined from MR spectra is 2.09 and 1.49 eV, respectively. It gives the energy separation of 0.70 and 0.28 eV between the direct and indirect gap in HfS_2_ and HfSe_2_. Significantly larger band gap values determined from MR mean that HfS_2_ and HfSe_2_ are indirect gap semiconductors. The found indirect gaps are consistent with previous absorption studies for these crystals^47^, see Table 1. Extended optical studies will be reported elsewhere.

The next couple of TMDs are WX_2_ (X = S and Se) compounds, which are quite intensively studied in recent years^14,19–21,23^, and are known as indirect gap semiconductors in their bulk form, but the value of the indirect gap is not well established for these compounds. The band gap determined from PA measurements for WS_2_ and WSe_2_ is 1.57 and 1.33 eV, respectively. The energy of A transition for WS_2_ and WSe_2_ is 2.05 and 1.65 eV, respectively. It gives the energy separation of 0.48 eV (WS_2_) and 0.32 eV (WSe_2_) between the direct and indirect gap for this couple of crystals. Energies of direct optical transitions observed in MR (A, A_H_, and B transitions) are in good agreements with previous studies for these compounds^39^.

For the last couple of TMDs (ReS_2_ and ReSe_2_) the separation between energy gaps determined from PA and MR is significantly smaller. For ReS_2_ the band gap determined from PA and MR measurements is 1.37 and 1.55 eV, respectively, while for ReSe_2_ it is 1.18 eV (PA) and 1.31 eV (MR). These energies are very consistent with those founded by Ho *et al*.^12^, see Table 1, and mean that these bulk crystals have the indirect gap. The energy difference between the direct and indirect gap decreases from 0.18 to 0.03 eV with replacing S by Se atoms.

By using PA and MR spectroscopy it has been concluded that for all these couples of TMD replacing S atoms by Se atoms leads to crystals with larger lattice constant and narrower band gap (both indirect and direct) as well as smaller energy difference between band gap types. During the analysis of the influence of transition metal atoms exchange on the indirect and direct band gap in TMD it should be noted that the change in the transition metal also leads to a change in the crystallographic structure. HfX_2_ crystals can have a 1 T structure with octahedral coordination of the chalcogenides by the metal atom^47^, WX_2_ samples are the hexagonal 2 H layered crystals like MoX_2_ samples, and ReX_2_ samples have a distorted 1 T diamond-chain structure with the triclinic symmetry (3 R polytype)^1,14^. The configuration of the transition metal in this case is 5d^2^6s^2^, 5d^4^6s^2^, and 5d^5^6s^2^ for Hf, W, and Re, respectively. As seen, the difference is only in d electrons but it is important enough to prefer different crystallographic structures. Because of this the Brillouin zone and hence the electronic band structure for the three crystals vary significantly. Moreover, it is worth noting that various polytypes can coexist in the same TMD crystal, see for example recent studies for MoS_2_^48^. In our opinion it can be used as an explanation of various indirect gaps reported for the same bulk TMD crystals in recent years.

Summarizing the analysis of chemical trends it is also worth noting that the mixing of group VI atoms (S, Se, Te) in the studied TMDs should lead to alloys with the same crystallographic structure while the mixing transition metal atoms (e.g. YS_2_ with Y = Hf, W, Re) can be associated with a crystallographic phase transition at a given content and this issue is still unexplored for some of TMD alloys.

**Figure 4:**
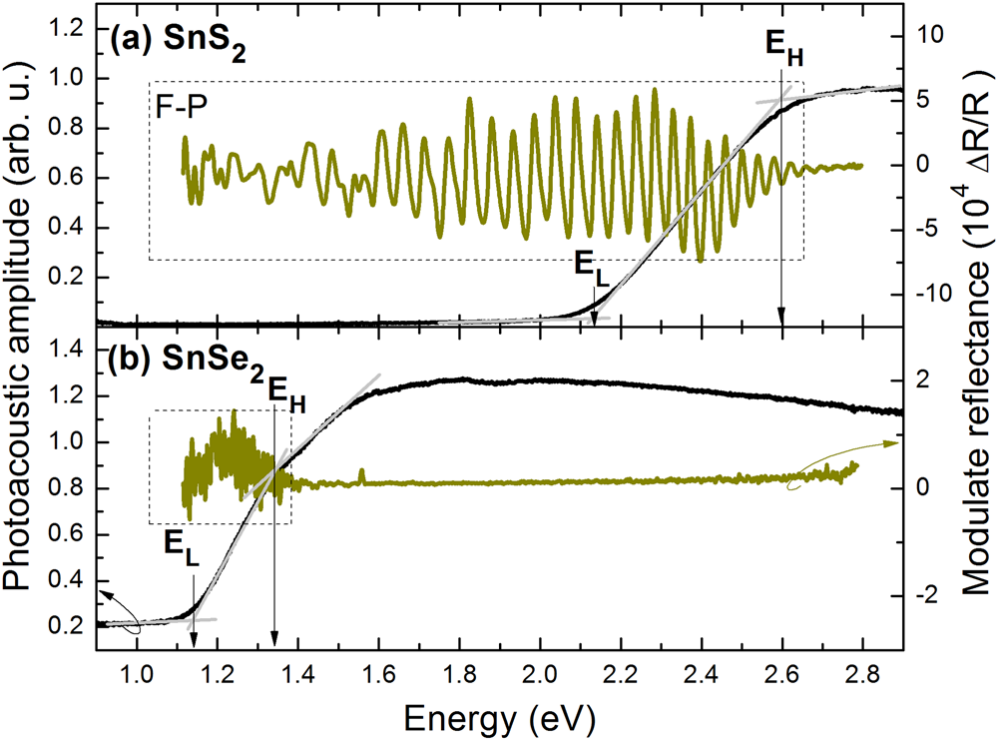
Room temperature photoacoustic spectra (black line) and modulated reflectance spectra (dark green line) of (**a**) SnS_2_ and (**b**) SnSe_2_ crystals.

The last couple of dichalcogenides includes SnS_2_ and SnSe_2_. In general, these compounds were quite intensively studied in different contexts in recent years but no reports on PA or MR measurements were published for these materials so far. Figure 4 shows PA and MR spectra for SnS_2_ and SnSe_2_ sample measured at room temperature. The band gap extracted from PA spectrum is 2.36 and 1.24 eV for SnS_2_ and SnSe_2_, respectively. These values are consistent with the expected chemical trend for these compounds, as well as with MR spectra where a strong Fabry-Perot (F-P) oscillations are observed. In general, F-P oscillations can be observed in MR spectra in the transparency region of the investigated sample^49,50^. Such signal is also observed for some previous TMD samples, see dashed boxes in Figs 2 and 3. It is worth to state that it cannot be attributed to any intrinsic optical transition since its intensity and period depend on the sample thickness, contrast of the refractive index, and other parameters. However, the observation of such signal can be used to evaluate the value of indirect gap in the investigated sample since this signal disappears (or its intensity is significantly reduced) above the indirect gap. For SnS_2_ and SnSe_2_ samples the F-P signal disappears at the band gap energy determined from PA measurements. Since no photoluminescence was observed for these samples we can conclude that for these samples the band gap has the indirect character. Moreover, in the studied spectral range (E < 2.8 eV) we did not observe any signal in MR spectra which could be attributed to a direct optical transition in these compounds. It can be associated with the fact that direct optical transitions for these compounds are expected at higher energies and/or the optical quality of the investigated samples is not high enough. In MR spectroscopy a modulation of built-in electric field is required. Such modulation can be strongly suppressed for poor quality samples while a heat generation, which is necessary for PA measurements, is not affected by weak material quality favoring PA spectroscopy in this case.

Among monochalcogenides, we have measured PA and MR spectra for III-VI (GaS, GaSe, and InSe) and IV-VI (GeS and GeSe) compounds. In general, these compounds were studied for a long time and MR spectra were measured for some of them (GaS, GeS, and InSe)^42,44^, but PA spectroscopy was applied to study only InSe^51^. Therefore, it is interesting to show how PA spectroscopy works for this material group and determine the band gap for these compounds utilizing the previously proposed routine.

**Figure 5:**
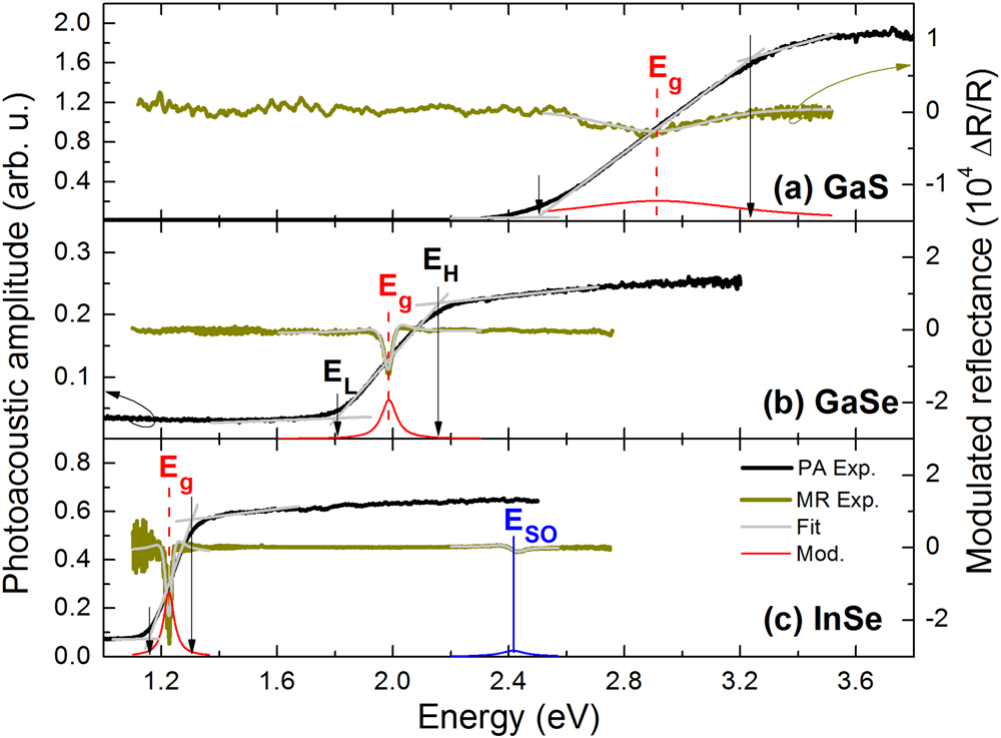
Room temperature photoacoustic spectra (black line) and modulated reflectance spectra (dark green line) of (**a**) GaS, (**b**) GaSe, and (**c**) InSe crystals.

Figure 5 shows PA and MR spectra measured at room temperature for GaS, GaSe, and InSe samples. The band gap determined from PA measurements is equal to 2.87, 1.98, and 1.23 eV for GaS, GaSe, and InSe, respectively. For GaSe and InSe this energy and the energy of the direct optical transition observed in MR spectra are the same within the experimental uncertainty. It means that these compounds can be classified as direct gap semiconductors. This conclusion is also confirmed by photoluminescence (PL) measurements since a bandgap-related emission was observed for both samples. It is worth noting that PL for such crystals was reported by other authors, see for example refs^52–55^, and therefore emission properties are not further discussed in this work. In addition, the MR spectrum observed for InSe is very consistent with previous photoreflectance studies of γ-InSe^56^. The bulk InSe sample studied in this work is also γ-polytype (i.e., 2H polytype according to the nomenclature used in this work) and extended studies on exfoliated layers from such crystal can be found in refs^54,55^. The oscillating signal below the band gap is due to F-P oscillations with a short period. The observed E_SO_ transition at ~2.4 eV is related to the direct optical transition between the spin-orbit split-off band and the conduction band. This sample is an example of high quality direct gap semiconductor where the band gap determined from PA and its broadening correspond to the direct optical transition and its broadening observed in MR.

For GaS no band gap related PL was observed. The band gap determined from PA measurements is 0.05 eV lower than the direct optical transition observed in the MR spectrum. In this case the MR resonance is broadened few times more than the MR resonance which is observed for GaSe and InSe, see Fig. 5. It can be associated with worse quality of this sample but also with some intrinsic property of this compound since a large increase of the broadening of MR resonance with the increase in temperature was observed for this compound in piezoreflectance spectra, see ref.^44^.

In the context of previous discussion of chemical trends for the energy gap, these compounds also behave like III-V or II-VI compounds: replacing small atoms by larger atoms from group VI (compare GaS and GaSe) or from group III (compare GaSe and InSe) the lattice constant increases and the band gap narrows. It is also worth noting that various polytypes monochalcogenides complicate studies of bulk crystals and therefore comparison with the literature is problematic since poor information about crystallographic structure is provided in many cases. However, the direct band gap values determined by PA and MR agree very well with the previous studies of these compounds, see Table 1.

**Figure 6:**
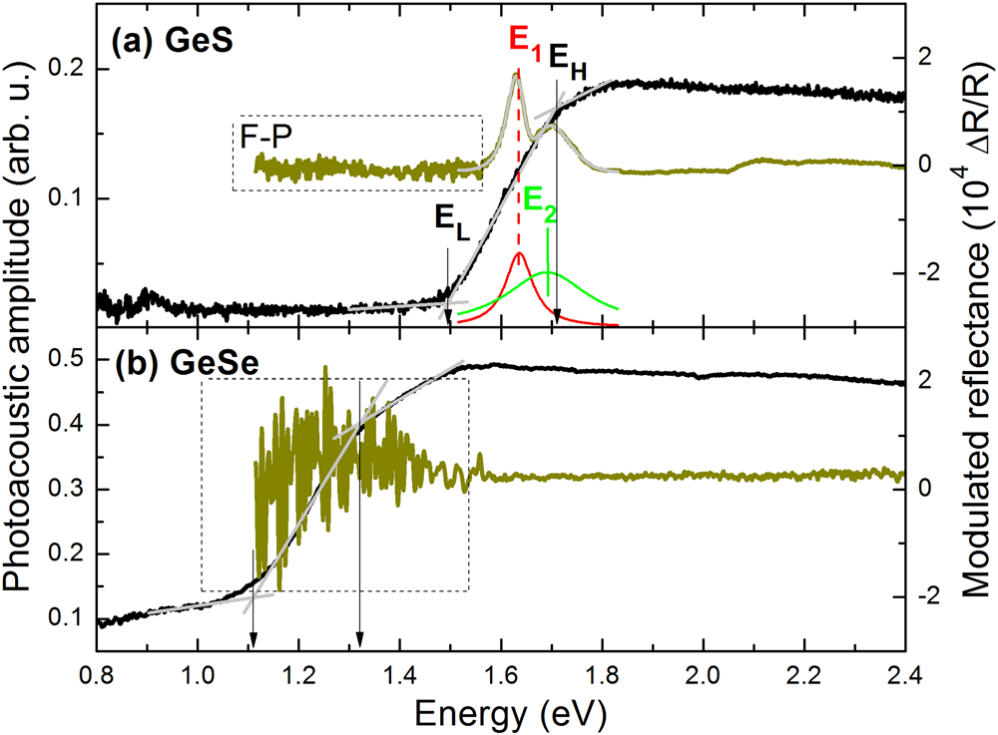
Room temperature photoacoustic spectra (black line) and modulated reflectance spectra (dark green line) of (**a**) GeS and (**b**) GeSe crystals.

Figure 6 shows PA and MR spectra measured for GeS and GeSe samples. In this case no PL was observed and therefore an indirect character of band gap can be expected. The band gap determined from PA measurements for GeS and GeSe sample is 1.60 and 1.22 eV, respectively. For GeS two direct optical transitions are observed (E_1_, E_2_) near the band gap energy determined from PA measurements but their spectral position is at higher energies (1.64 and 1.69 eV). It might mean that GeS is an indirect gap semiconductor. No optical transition in MR spectrum of GeSe can be associated with poor quality of this crystal or no band bending modulation in this sample. Energies found for this couple of compounds are qualitatively consistent with recent theoretical studies of energy gap for these crystals, i.e., replacing smaller S atoms by larger Se atoms the band gap narrows, following the well-known trend in standard semiconductors^57–59^. The quantitative agreement with the previous experimental studies^58^ is also quite good, see Table 1. Extended optical studies of GeS will be reported elsewhere.

The graphical method to determine the band gap from PA measurements proposed in this work can be treated as the first step to conclude about the character of the energy gap if PA measurements are compared with MR measurements. For comparison, another approach can be applied. In semiconductor materials the optical absorption coefficient (*α*) in the vicinity of the absorption edge varies with the photon energy (*hν*) according to the expression

1 $$\alpha (h\nu )={\alpha }_{0}{(\frac{h\nu -{E}_{g}}{{E}_{g}})}^{1/n}$$

where *α*_0_ and *E*_*g*_ are a constant and the band gap energy, respectively, and *n* is a parameter characterizing the transition process: *n* = 2 for a direct transition and *n*=1/2 for an indirect transition. Using the proper value of *n* parameter we plot ${A}^{n}(h\nu )$ as a function of photon energy, *hν*, where *A* is the PA signal intensity, and it is assumed that *A* is proportional to the absorption coefficient ($A\propto \alpha$) in the range of absorption edge. The linear extrapolation of ${A}^{n}(h\nu )$ to 0, allows a direct determination of the band gap energy *E*_*g*_, as shown in our previous paper on GaAs(Bi) nanowires^38^. This approach was not introduced previously in this paper for clarity, since the *n* parameter is rather not obvious for many compounds studied in this work. However, further studies of particular van der Waals crystals by PA spectroscopy can utilize Eq. (3) with the proper *n* parameter.

**Figure 7:**
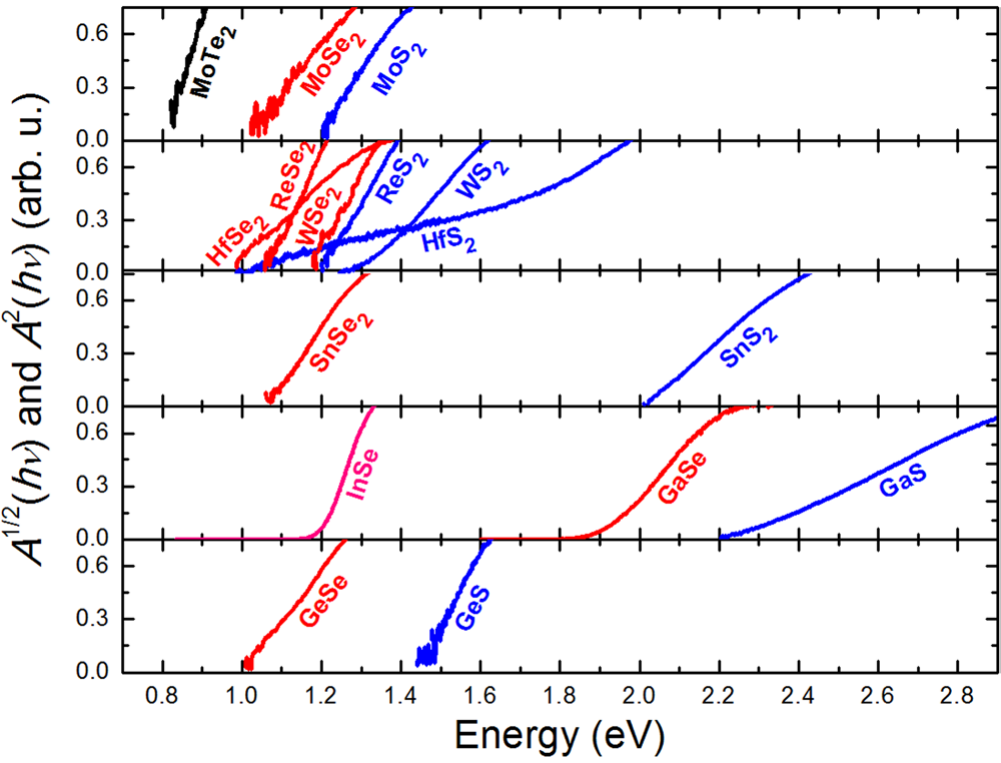
Plots of ${A}^{1/2}(h\nu )$ and ${A}^{2}(h\nu )$ versus the photon energy used to determine the absorption edge for van der Waals crystals with indirect and direct gap, respectively (direct gap semiconductors are InSe and GaSe). *A* is the amplitude of PA signal. vdW crystals containing S and Se atoms are represented by blue and red lines, respectively. Black and rose lines correspond to MoTe_2_ and InSe, respectively.

Figure 7 shows the analysis of PA spectra (i.e., ${A}^{n}(h\nu )$ plot with *n* = 2 and 1/2 for direct and indirect gap, respectively) in the range of absorption edge for all sets of samples studied in this work. The band gap values determined from these plots are given in Table 1. In addition, the E_L_ energy, which corresponds to the low energy side absorption, is given in Table 1. For indirect gap semiconductors the band gap determined with formula: ${E}_{g}=({E}_{L}+{E}_{H})/2$ can be overestimated due to small absorption coefficient in the spectral range of indirect absorption. This range is easy to estimate if the direct optical transition is observed in MR spectra since it is the energy difference between the direct and indirect band gap (ΔE) which is given in Table 1. This difference correlates very well with the broadening of indirect gap determined from formula: ${{\rm{\Gamma }}}_{g}=({E}_{H}-{E}_{L})/2$, i.e., larger broadening is observed for compounds with larger ΔE. It is consistent with principles of PA signal generation for indirect gap semiconductors. Thus, in this case the band gap determined with Eq. (3) is the most accurate which corresponds to the indirect edge in the studied compounds. For direct gap semiconductors the situation is different due to large absorption coefficient and therefore the broadening of direct optical transition observed in MR spectrum is comparable with the broadening of absorption edge determined from PA measurements. It means that the comparison of the broadening of optical transitions observed in PA and MR spectra is the next criteria for the evaluation of the band gap character in the studied compound. The proposed analysis routine of PA spectra via determination of E_L_ and E_H_ energies by the knee method has been proven to be useful in the interpretation of combined PA and MR spectra.

**Figure 8:**
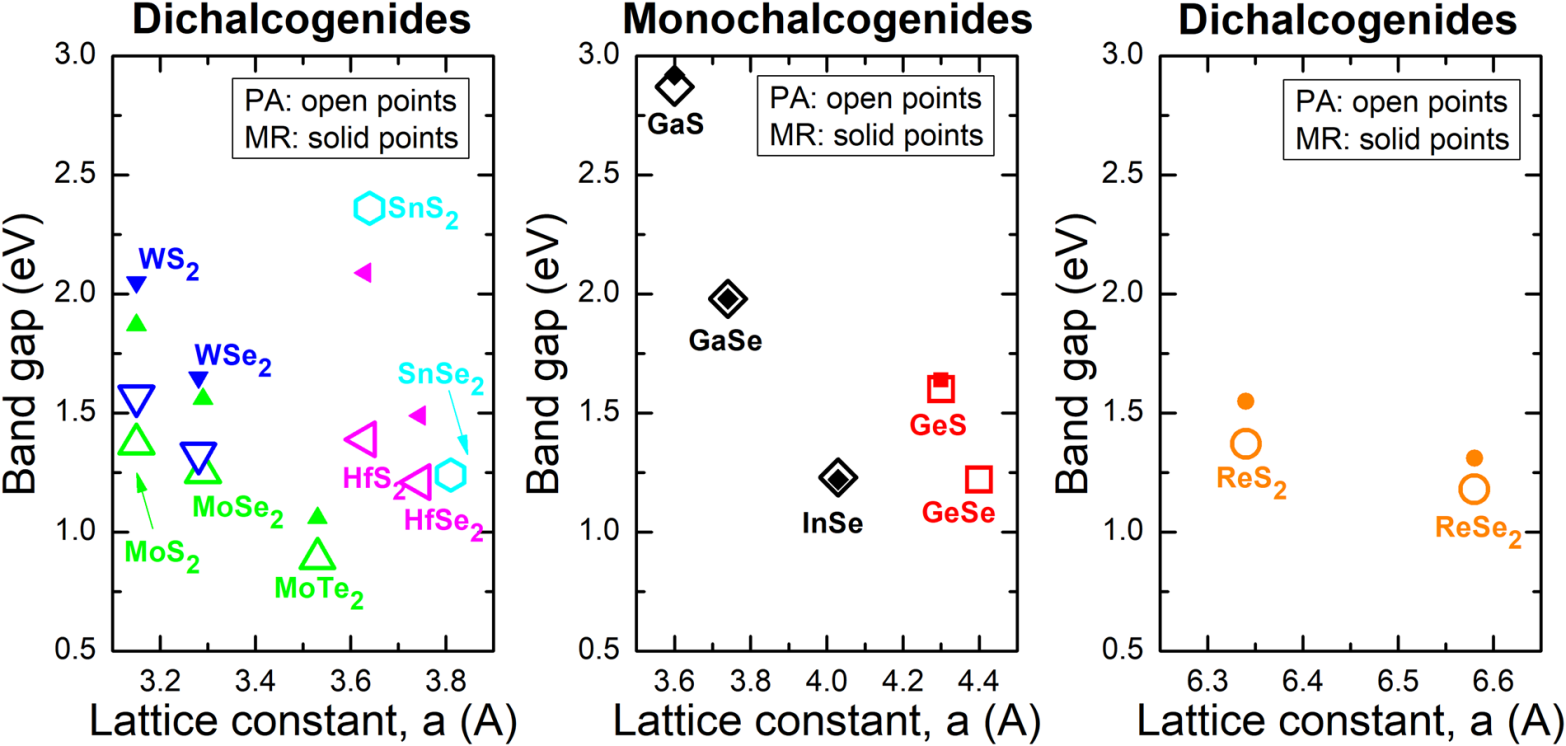
Band gap determined from PA (open points) and MR (solid points) measurements at room temperature and plotted versus the lattice constant. Lattice constants were taken from sample manufacturers’ websites, except for InSe where the value was taken from ref.^62^. Error bars are smaller than sizes of points.

Chemical trends observed for indirect and direct gap for the studied van der Waals crystals are summarized in Fig. 8. In this figure a decrease of indirect and direct gap with the increase in the lattice constant it is clearly visible for the set of crystals where chalcogenide atoms (S, Se, and Te) are changing. In this case no change in the crystallographic structure is observed and therefore this situation can be compared with III-V or II-VI compounds. A change in the crystallographic structure takes place when metal atoms (e.g., Hf, W, and Re) are changing and therefore band gaps for such sets of van der Waals crystals are not discussed in the context of chemical trends typical of semiconductor compounds and alloys.

## Conclusions

It has been shown that PA spectroscopy is a powerful tool to study the indirect band gap in van der Waals crystals. An important advantage of this method over the regular absorption measurement, which can be used to determine the indirect gap, is that PA spectroscopy can be applied to very thin layers where the light absorption is weak and hard to detect, as well as to samples which scatter the incident light. Another important advantage of PA spectroscopy over photocurrent spectroscopy, which is another method to determine the indirect gap, is its contactless character which does not need fabrication of electric contacts on the sample. In this work the band gap values have been determined for various van der Waals crystals by analyzing the PA spectra and the character of the band gap (indirect vs. direct) has been identified based on the comparison between PA and MR spectra. For this purpose, a proper analysis of PA spectra has been proposed in this work. Since MR probes only direct optical transitions, it was possible to conclude about the character of the energy gap observed in PA spectra and the energy difference between the indirect and direct band gap in the studied samples. It has been observed that both indirect and direct band gap in proper sets of vdW crystals follow the well-known chemical trends in semiconductor compounds: i.e., the lattice constant increases and both energy gaps narrow while increasing the atom size. In addition, it has been shown that the energy difference between the direct and indirect gap decreases with the replacing small atoms by larger atoms in proper sets of vdW crystals.

## Methods

### Photoacoustic measurements

PA spectra were measured using the gas-microphone method originally proposed by Rosencwaig^60^. The light from a 250 W halogen lamp with the spectral distribution corresponding to a black-body radiation (3550 K) was dispersed with a 0.32 m focal length monochromator and modulated using mechanical chopper. The spectral width of light after the monochromator was estimated to be ~2–4 nm. The monochromatic light was focused on the sample enclosed inside a non-resonant photoacoustic cell made of aluminum. The illumination spot size was about 2 × 5 mm and the light intensity was estimated to be from 1–10 μW range depending on the wavelength. The acoustic waves generated inside the cell were detected with an electret condenser microphone (sensitivity 20 mV/Pa) producing the AC voltage signal further demodulated using a lock-in amplifier (Stanford Research Systems SR830). Measurements were performed with an integration time of 3 seconds and using the AC input coupling. Typical signal amplitudes obtained during the measurements (in the saturation region) were in the range of 10–50 mV taking into account the amplification of 200 V/V (SR560 voltage amplifier). All spectra were normalized with respect to the reference spectrum obtained for powdered carbon and the PA signal is given in arbitrary units. This normalization procedure assumes that the absorption of powered carbon is spectrally flat and thereby the spectral characteristic of the light source influenced by optical elements (diffraction grating, lenses, and filters) can be determined. All PA measurements were performed at room temperature (295 K).

### Modulated reflectance measurements

For photoreflectance measurements a single grating 0.55 meter focal-length monochromator and a Si (or an InGaAs) *pin* photodiode were used to disperse and detect the light reflected from the samples. The spectral resolution of this system was below 1 nm. A 150 W tungsten-halogen bulb with the spectral distribution corresponding to a black-body radiation (3450 K) was used as the probe, and a semiconductor laser (405 nm line) was used as the pump source. The pump beam was modulated by a mechanical chopper at a frequency of 280 Hz. Phase-sensitive detection of the PR signal was performed using a lock-in amplifier (SR830). The lock-in parameters were the same as for photoacoustic measurements. Other relevant details on PR measurements can be found in ref.^29^. All MR measurements were performed at room temperature (295 K). To determine the energy of direct optical transitions observed in MR spectra, these spectra have been fitted using Aspnes formula^61^, which is widely applied to analyze MR spectra^39,46^. This formula is given by Eq. (2)

2 $$\frac{{\rm{\Delta }}R}{R}(E)={\rm{Re}}[C{e}^{i\vartheta }{(E-{E}_{j}+i{\rm{\Gamma }})}^{-m}],$$

where $\frac{{\rm{\Delta }}R}{R}(E)$ is the energy dependence of MR signal, C and ϑ are the amplitude and phase of the line, and E_j_ and Γ are the energy and the broadening parameter of the optical transition, respectively. The term m is assumed to be 2, which corresponds to the excitonic transition. The moduli of MR resonance have been obtained according to Eq. (3)

3 $${\rm{\Delta }}\rho (E)=\frac{|C|}{{[{(E-{E}_{j})}^{2}+{{\rm{\Gamma }}}^{2}]}^{\frac{m}{2}}}$$

with parameters taken from the fit and plotted by a dashed line next to the MR spectrum.

### Samples preparation

Samples from 2Dsemiconductors Inc. (MoS_2_, HfS_2_, HfSe_2_, WS_2_, SnS_2_, SnSe_2_, and GeSe) and HQ Graphene company (MoSe_2_, MoTe_2_, WSe_2_, ReS_2_, ReSe_2_, GaS, GaSe, and GeS) were used to study the indirect and direct band gap by PA and MR spectroscopy. All of the samples were grown using the Bridgman technique. No special sample treatment was done prior to the measurements. Samples used for PA and MR measurements were macroscopic sizes (~3 × 3 mm with thickness varying from 0.1 to 0.5 mm) and their purity represents the present state of the art at above companies (>99.99%).

The datasets generated during the current study are available from the corresponding author on reasonable request.
